# Cerebral Venous Sinus Thrombosis following Diagnostic Curettage in a Patient with Uterine Fibroid

**DOI:** 10.1155/2014/270654

**Published:** 2014-06-01

**Authors:** Xiao-Qun Zhu, Li Cao

**Affiliations:** Department of Neurology, The First Hospital of Anhui Medical University, Hefei 230022, China

## Abstract

Cerebral venous sinus thrombosis (CVST) is a relatively rare cerebrovascular disease, of which the risk has been documented in patients with numerous conditions. However, CVST has never been previously described in association with the use of a diagnostic curettage in patient with uterine fibroid. Herein, we described a 43-year-old woman who presented with recurrent convulsive seizures and severe and progressive headache 1 day after a diagnostic curettage of the uterus, which was confirmed to be uterine fibroid pathologically later, and her condition subsequently progressed to confusion. Brain magnetic resonance imaging (MRI) revealed an acute extensive thrombosis of the left transverse and sigmoid sinus and the ipsilateral cerebellum infarction. Evaluation for primary thrombophilia revealed that an iron deficiency anemia (IDA) due to the fibroid bleeding induced menorrhagia together with a diagnostic curettage might be the sole hypercoagulable risk factor identified. Treatment with anticoagulation led to full recovery of her symptoms and recanalization of the thrombosis was proven on magnetic resonance venography (MRV) 2 months later. We suggest that CVST should be recognized as a potential complication related to this diagnostic technique, especially in patient with IDA. The early diagnosis and timely treatment would be of significance in improving the prognosis of this potentially lethal condition.

## 1. Background

Cerebral venous and sinus thrombosis (CVST) is a relatively rare condition with an incidence of about 7 per 1000000 and a mortality rate of between 10 and 20%. Females are more commonly affected than males with a ratio of approximately 3 to 1 in young to middle-aged adults [1]. This skewed gender ratio of CVST incidence is usually attributed to gender-specific risk factors, for example, oral contraceptives, extent pregnancy, puerperium, and hormone replacement therapy, and these conditions are consistently present in case series [2]. Many other conditions appear only in anecdotal reports, such as fibrous thyroiditis, surgery, head trauma, paraneoplastic syndrome, and autoimmune disease [3], and a causal link between these conditions and CVST is pending to be determined. Iron deficiency anemia (IDA) has occasionally been linked to CVST in several pediatric cases [4, 5] and in rare adult cases [6], though there is no direct evidence showing that IDA causes CVST. Herein, we report, for the first time, a CVST associated with the diagnostic curettage of the uterus in a patient with IDA and uterine fibroid.

## 2. Case Report

A 43-year-old woman with a two-day history of an increasing headache presented to our department with three episodes of tonic-clonic seizures over a two-hour period. The headache was described as sharp and located primarily in the bilateral frontal regions earlier and the whole brain later. Each episode of seizure would last 1 to 2 minutes. After admission, the headache symptom progressed more severely along with nausea and vomiting. She had no prior history of epilepsy or recurrent headaches, deep venous thrombosis, or other thrombotic events. She also denied recent head trauma. Her past medical history was otherwise unremarkable, but, three days prior to the admission, that is, one day before this event occurred, she had undergone a diagnostic curettage for uterine fibroid with no immediate complication, and the uterine fibroid was pathologically confirmed later. Her family history was unremarkable for any known thrombotic events.

On admission, the patient appeared to be delirious and confused during the interview. Her vital signs were as follows: temperature 36.8°C, pulse rate 86/minute, blood pressure 140/80 mm Hg, and respiration rate 20/minute. The physical examination did not reveal any abnormality in chest or in abdomen. The neurological examination revealed that her pupils were equal in size (diameter, 2 mm) and round in shape, and the pupillary light reflex was normal on both sides. The examination of the muscle strength of the limbs could not be completed as the patient was uncooperative, but the limb-associated spontaneous activity was observed. Tendon reflexes of the limbs were detected symmetrically weakened, and normal plantar reflexes were detected bilaterally. Mild stiffness of her neck was found.

**Figure 1:**
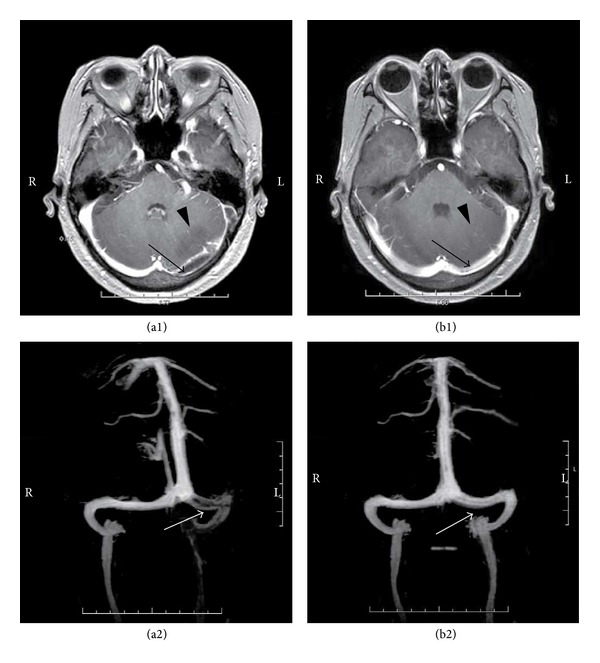
Brain MRI and MRV scan before and after treatment. MRV scan demonstrates loss of signal in the left transverse and sigmoid sinus, representing venous sinus thrombosis before treatment (indicated by arrows on (a1) and (a2)). The signal in the left transverse and sigmoid sinus recovered two months after the initiation of treatment (indicated by arrows on (b1) and (b2)), representing recanalization. MR image shows a patchy hypointense lesion in the left hemisphere of the cerebellum before treatment (indicated by arrow head on (a1)), and this patchy hypointense lesion recovered in the corresponding area after treatment (indicated by arrow head on b1).

Routine laboratory investigations were conducted immediately after her admission. Blood cell count indicated white blood cell count of 8.15 × 10^9^/L (normal range, 3.97–9.15 × 10^9^/L) and percentage of neutrophil to white blood cell of 70.80% (normal range, 51–75%). Other laboratory data revealed red blood cell count of 3.15 × 10^12^/L (normal range, 3.8–5.1 × 10^12^/L), hypochromic microcytic anaemia with an initial haemoglobin (HGB) value of 76 g/L (normal range, 131–172 g/L), a mean corpuscular volume (MCV) of 71 fL (normal range, 83.90–99.10 fL), hematocrit of 28% (normal range, 35–45%), and a platelet count of 260 × 10^9^/L (normal range, 85–303 × 10^9^/L). Serum iron concentration was 32 *μ*g/dL (normal range, 90–190 *μ*g/dL), ferritin concentration 9.8 ng/mL (normal range, 10–120 ng/mL), and total iron-binding capacity 74 *μ*mol/L (normal range, male 50–77 *μ*mol/L, female 54–77 *μ*mol/L). Other laboratory tests including random blood sugar, urea, creatinine, sodium, potassium, aspartate aminotransferase, alanine aminotransferase, and alkaline phosphatase yielded normal. The coagulation profile was within normal range, the prothrombin time was 12 seconds, partial thromboplastin time was 27.6 seconds, and INR was 1.1. A computed tomography (CT) of the head excluded intracerebral hemorrhage on the day the headache occurred. Lumber puncture was carried out after mannitol administration on the second day after admission and showed clear liquid with 400 mm H_2_O of pressure. Laboratory tests of CSF revealed the following results: protein 0.59 g/L (normal range, 0.15–0.45 g/L), chloride 119.6 mmol/L (normal range, 120–130 mmol/L), glucose 2.60 mmol/L (normal range, 2.5–4.4 mmol/L), white blood cell count 3 × 10^6^/L (normal range, 0–5 × 10^6^/L), and red blood cell (RBC) count 0 × 10^6^/L (normally no RBC). A magnetic resonance venography (MRV) demonstrated extensive thrombosis in the left sigmoid and transverse sinuses, and a cerebellar infarction in the territory of the left sigmoid and transverse sinuses (Figure 1(a)), confirming a diagnosis of CVST.

The seizures were controlled with intravenous diazepam and a loading dose of intravenous sodium valproate. On the other hand, the patient was treated with transfusion of packed red blood cells and oral iron supplementation. After the diagnosis was confirmed by MRV, anticoagulation with low molecular weight heparin (LMWH) was initialized and maintained for 2 weeks and followed by oral warfarin. The patient's headache and ataxia had recovered completely by the end of the second month. Two months after the symptom onset, a repeated MRV revealed recanalization of the left sigmoid and transverse sinuses (Figure 1(a)).

## 3. Discussion

CVST is a rare but potentially fatal disease marked by clotting of blood in cerebral veins or dural sinuses. It was previously related to the infections of mastoid, orbit, and central face derma. However, due to the existence of modern effective antibiotics, it is more often related to pregnancy, puerperium, systemic diseases, dehydration, intracranial tumors, and oral contraceptives, and the most common causes are coagulopathies [3], such as essential thrombocythemia, dysfibrinogenemia, and systemic lupus erythematosus. Imaging studies remain to be the cornerstone for the diagnosis of CVST. The imaging modality of choice is MRV as it allows a direct visualisation of the large cerebral veins and the dural venous sinuses. Treatment options for CVST include anticoagulation, thrombolytic therapy, and, in some cases, surgical thrombectomy.

The thrombosis in our patient was in the left transverse and sigmoid sinus which are often associated with mastoiditis and otitis media or infection that spreads to sigmoid sinus through the neighboring eroded bone [2]. Our patient had no mastoiditis or otitis media and her body temperature, blood white cell and neutrophil counts, and the CSF cell counts were all in normal range. Thus, the CVST in our patient was not attributed to infection, which was further supported by the full recovery without using antibiotics. Numerous case reports have substantiated that uterine fibroids may, though seldom, induce deep vein thrombosis (DVT). Statistical analysis from case cohort has shown that the incidence of DVT is significantly higher in patients whose uterine weight is 1,000 gm or more [7], indicating that DVT is due to the vascular stasis created by the compression of the veins in the pelvis by the enlarged uterus, but not due to the coagulatory changes induced by the fibroid. On the other hand, uterine fibroid can be associated with secondary polycythemia [8] which may have a potential complication of CVST [2], but our patient had no polycythemia. Thus, the intracranial thrombosis in our patient was caused through neither the mechanism of the uterine fibroid per se nor the mechanism of polycythemia related to the uterine fibroid.

In our patient, the sole potential hypercoagulable risk factor identified was the iron deficiency anemia (IDA) which has been linked to the occurrence of cerebral venous thrombosis (CVT) or CVST in infants or adults by several case reports [4–6]. And a prospective study from a case cohort of CVT patients also indicates that severe anemia is significantly and independently associated with CVT [9]. Three possible mechanisms are proposed to be involved in the development of thrombosis in patients with IDA. First, thrombocytosis secondary to IDA causes thrombosis. Normal iron levels are required to prevent thrombocytosis through inhibiting thrombopoiesis. Second, iron deficiency results in a hypercoagulable state because of reduced red cell deformability and increased viscosity. Third, under the condition of stress, anemic hypoxia created by the rises of metabolic demand at the tissue level can predispose to venous thrombosis. Our patient's IDA should be attributed to the menorrhagia due to the uterine fibroid bleeding, as she reported menorrhagia and longer time menstruation, and, in adults, the most common cause of IDA is blood loss resulting from menorrhagia in women, chronic gastrointestinal disease (ulcer, malignancy, and parasites), hemoglobinuria, and hemoptysis. This is consistent with a previous report that CVST developed in two patients who had IDA due to uterine fibroids [10]. But the CVST in our patient did not occur until the time the uterine curettage was conducted though the fibroid and ICA existed there for long time. Thus, uterine curettage may act as a trigger of CVST for patients who have already been in a condition of thrombophilia. For instance, cesarean delivery is shown to be an independent risk factor that has a significant coefficient used to multiply the basic risk of developing CVST [3]. Thus, we speculate that, in our patient, uterine curettage may also act as a factor coefficient with the IDA leading to the formation of CVST.

For the treatment of CVST, supportive or symptomatic measures, including hydration, seizure control with anticonvulsants, intracranial pressure control, and appropriate antibiotics, are usually taken. In our patient, antibiotics were not employed as no infection was detected based on the normal CSF and blood white cell counts. Full anticoagulation therapy (ACT) shall be initialized to recanalize and to prevent further spread of thrombus. Unfractionated heparin or low molecular weight heparin (LMWH) is usually recommended in the absence of any major hemorrhage. The duration of heparin administration ranges from several days to three weeks. Subsequently, administration of oral anticoagulant is recommended, with treatment duration of 3–6 months depending on the venous imaging to evaluate recanalization and resolution of clot. Our patient was kept on anticoagulation therapy with LMWH for 2 weeks followed by oral warfarin.

In summary, we described the complication of CVST after a diagnostic curettage for uterine fibroid in a patient with IDA but no other obvious risk factors associated with thrombosis. Uterine curettage per se may act as a factor coefficient with other conditions associated with CVST. Progressing headache or in combination with seizures in patients with uterine fibroids after uterine curettage should raise suspicion for CVST. The early diagnosis and timely treatment would be of significance in improving the prognosis of this potentially lethal condition.
